# COVID-19 Impact on DTP Vaccination Trends in Africa: A Joinpoint Regression Analysis

**DOI:** 10.3390/vaccines11061103

**Published:** 2023-06-15

**Authors:** Ines Aguinaga-Ontoso, Sara Guillen-Aguinaga, Laura Guillen-Aguinaga, Rosa Alas-Brun, Luc Onambele, Enrique Aguinaga-Ontoso, Francisco Guillen-Grima

**Affiliations:** 1 Department of Health Sciences, Public University of Navarra, 31008 Pamplona, Spain; sguillen.4@alumni.unav.es (S.G.-A.); lauraguillencole@gmail.com (L.G.-A.); rosamaria.alas@unavarra.es (R.A.-B.); 2 Healthcare Research Institute of Navarra (IdiSNA), 31008 Pamplona, Spain; 3 Sykepleieavdelingen, Suldal Sykehjem, 4230 Sand, Norway; 4 School of Health Sciences, Catholic University of Central Africa, Yaounde 1110, Cameroon; onambele.luc@ess-ucac.org; 5 Department of Sociosanitary Sciences, University of Murcia, 30120 Murcia, Spain; aguinaga@um.es; 6 Department of Preventive Medicine, Clínica Universidad de Navarra, 31008 Pamplona, Spain

**Keywords:** DTP vaccine, Africa, COVID-19, vaccine coverage, joinpoint regression, healthcare system, vaccination rates

## Abstract

Background: Deaths due to vaccine-preventable diseases are one of the leading causes of death among African children. Vaccine coverage is an essential measure to decrease infant mortality. The COVID-19 pandemic has affected the healthcare system and may have disrupted vaccine coverage. Methods: DTP third doses (DTP3) Vaccine Coverage was extracted from UNICEF databases from 2012 to 2021 (the last available date). Joinpoint regression was performed to detect the point where the trend changed. The annual percentage change (APC) with 95% confidence intervals (95% CI) was calculated for Africa and the regions. We compared DTP3 vaccination coverage in 2019–2021 in each country using the Chi-square test. Result: During the whole period, the vaccine coverage in Africa increased with an Annual Percent change of 1.2% (IC 95% 0.9–1.5): We detected one joinpoint in 2019. In 2019–2021, there was a decrease in DTP3 coverage with an APC of −3.5 (95% −6.0; −0,9). (*p* < 0.001). Vaccination rates decreased in many regions of Sub-Saharan Africa, especially in Eastern and Southern Africa. There were 26 countries (Angola, Cabo Verde, Comoros, Congo, Côte d’Ivoire, Democratic Republic of the Congo, Djibouti, Ethiopia, Eswatini, The Gambia, Guinea-Bissau, Liberia, Madagascar, Malawi, Mauritania, Mauritius, Mozambique, Rwanda, Senegal, Seychelles, Sierra Leone, Sudan, Tanzania, Togo, Tunisia, Uganda, and Zimbabwe) where the vaccine coverage during the two years decreased. There were 10 countries (Angola, Cabo Verde, Comoros, Democratic Republic of the Congo, Eswatini, The Gambia, Mozambique, Rwanda, Senegal, and Sudan) where the joinpoint regression detected a change in the trend. Conclusions. COVID-19 has disrupted vaccine coverage, decreasing it all over Africa.

## 1. Introduction

Vaccination is a pivotal public health intervention, substantially reducing morbidity and mortality associated with infectious diseases [1,2,3]. Immunization programs have contributed to a significant decline in the global burden of vaccine-preventable diseases, thus enhancing millions of individuals’ overall quality of life [4,5,6]. The diphtheria-tetanus-pertussis (DTP) vaccine plays an essential role in childhood immunization programs, safeguarding against three major infectious diseases that can lead to severe illness and potential death in children [7]. In 2012, the World Health Organization (WHO) introduced the Global Vaccine Action Plan (GVAP) 2011–2020 to endorse routine vaccination for all children worldwide [8]. Achieving high vaccination coverage is crucial to protect children and facilitate herd immunity.

Consequently, the WHO established a 90% vaccination coverage target for three doses of DTP (DTP3) by 2015. However, despite progress in recent years, achieving vaccine coverage remains a formidable challenge in many African countries [9]. Resource constraints, inadequate infrastructure, and sociocultural factors contribute to persistent disparities in vaccine access [10]. As per the WHO, vaccine-preventable diseases are a leading cause of child mortality, with over 300,000 pertussis-induced deaths reported annually [11]. Across different countries, vaccine coverage varies with factors such as urban versus rural residence, wealth status, education level, and frequency of maternal prenatal visits [12].

The COVID-19 pandemic has exerted unprecedented pressure on global healthcare systems, potentially affecting routine immunization programs [13,14,15]. The crisis has led to disruptions in vaccine supply chains, diversion of healthcare resources to manage the outbreak, and increased reluctance among communities to visit healthcare facilities due to fear of infection and vaccine hesitancy [16,17,18,19,20,21]. These challenges might have led to a decline in routine immunization coverage, putting millions of children at risk of vaccine-preventable diseases [22]. Understanding the magnitude of this disruption is critical for informing public health policy, formulating intervention strategies, and preparing for future sanitary crises [23]. Furthermore, it is essential to safeguard the health and well-being of children in Africa. A study reported global reductions of 7.7% and 7.9% in the coverage of DTP3 and the first dose of the Measles-Containing Vaccine (MCV1), respectively [24]. However, this study’s limitation is that it only includes data up to December 2020. Our study utilizes the percentage of infants who received the third DTP vaccine (DTP3) as the primary indicator for DTP vaccination coverage. As per the WHO, an unimmunized child is one who is between 12 and 23 months old and has not received DTP3 [25]. This metric is a reliable and widely accepted measure of the performance of an immunization program [23,26].

The administration of the third dose of the DTP vaccine is crucial as it completes the primary immunization series and ensures optimal protection against diphtheria, tetanus, and pertussis [27]. By focusing on this indicator, we can assess the effectiveness of immunization programs in reaching their target populations. The coverage rates of the first and third doses of the DTP vaccine serve as vital markers in gauging the efficacy of an immunization program. DTP1 coverage is often used to assess accessibility, reflecting the extent of health services’ reach and the program’s initial engagement with the population [28]. In contrast, DTP3 coverage demonstrates the program’s sustainability and effectiveness in ensuring that the target population receives all necessary doses [29]. A meta-analysis estimated that the prevalence of vaccination dropout in Africa was 26.06%, with Nigeria recording the highest immunization dropouts (33.59%) [30]. Discrepancies between DTP1 and DTP3 coverages can reveal issues with patient retention, hinting at potential barriers such as cost, transportation, and health education. Therefore, both metrics offer valuable insights for fortifying immunization programs [31].

Based on available data, the COVID-19 pandemic has significantly impacted DTP vaccination trends worldwide. According to the WHO and UNICEF, there has been a substantial decline in childhood vaccinations globally [32]. The proportion of children receiving DTP3 globally decreased by 5% between 2019 and 2021, representing the most significant decline in the past 30 years. In 2021, more than 25 million children worldwide missed one or more doses of DTP, over 6 million more than in 2019. UNICEF reports that vaccine coverage fell in every region, with the East Asia and Pacific region experiencing the most drastic decline in DTP3 coverage, dropping nine percentage points within just two years. The data from the CDC further illustrate the extent of this impact. In 2021, the estimated global coverage with three doses of diphtheria-tetanus-pertussis–containing vaccine (DTPcv3) decreased to 81%, marking the lowest point since 2008 [33]. Consequently, these children are at a heightened risk of developing vaccine-preventable infectious diseases.

This study aims to examine DTP3 vaccination coverage trends in Africa between 2012 and 2021, focusing on the impact of the COVID-19 pandemic. We hypothesize that the COVID-19 pandemic may have disrupted routine vaccination programs [34].

## 2. Materials and Methods

Vaccination rates were extracted from the UNICEF databases, which cover the period from 2000 to 2017, with the latest data update available in 2021 [35]. Western Sahara was excluded from the analysis due to its absence on the UNICEF database, while British and French territories were also excluded because they are not members of the African Union. We sourced regional estimations from the UNICEF database as well. As there were no estimations for North Africa, we calculated the DTP3 coverage estimation, weighted by population. Adhering to the African Union scheme, we included Algeria, Egypt, Libya, Mauritania, Morocco, and Tunisia in North Africa. However, we excluded the Sahrawi Arab Democratic Republic (Western Sahara) and South Sudan due to the lack of available data. The population of each country for every year of the study period was obtained from the World Bank [36].

We utilized joinpoint regression analysis, a widely recognized method for analyzing regional and country trends and detecting changes in various data types. This method allows the detection of periods of significant changes in incidence rates and quantification of the magnitude of change in each trend. Joinpoint regression is a statistical technique that identifies trend change points, termed “joinpoints.” It has been used previously in Africa to study the evolution of regional maternal mortality trends [37]. Employing joinpoint regression, the annual percentage change (APC) and the corresponding 95% confidence intervals (95% CI) were estimated to quantify the magnitude of change in each trend. In these models, vaccine coverage was the dependent variable, while the year was the independent variable.

We addressed autocorrelation in the time series data using the Durbin–Watson test [38]. Accounting for autocorrelation in our models offers several advantages, such as improved model fit due to recognizing dependencies within the series [39], reduced bias in parameter estimates, and adjusted significance levels, providing more accurate standard errors and confidence intervals. Nonetheless, it is important to note that employing autocorrelation models can result in more complex models, potentially leading to interpretation challenges and a risk of overfitting the data. This could compromise the model’s ability to generalize to new data or accurately represent the underlying trend.

We compared the DTP3 coverage for each country in 2019 with its coverage in 2021. A Paired Samples Test was performed to compare the means of DTP coverage in these two related groups, specifically for 2019 and 2021. We evaluated DTP3 vaccination coverage in Africa by country, comparing the coverage rates across different countries for 2019 and 2021. This analysis was conducted using the Chi-square test, wherein the coverage rates were weighted by the number of newborns in each country for the respective year. The data on the number of newborns per country per year were sourced from the UNICEF database [40].

All joinpoint computations were conducted using the joinpoint regression software (Joinpoint Regression Program, Version 4.9.1.0, National Cancer Institute, Bethesda, MD, USA). This software is a widely recognized tool for analyzing trends and detecting changes in various data types. It facilitated the effective implementation of joinpoint regression analysis and the assessment of autocorrelation in the time series data [41,42]. The joinpoint software automatically selects the number of joinpoints, using statistical tests to determine the optimal number. The statistical analysis comparing rates from 2019 to 2021 was conducted using IBM SPSS Statistics, version 26 (IBM Corp., Armonk, NY, USA). The data were checked for normality using the Shapiro–Wilk test. The level of significance was set at α = 0.05. The results were presented as the mean difference (MD) mean ± standard deviation (SD).

## 3. Results

**Figure 1:**
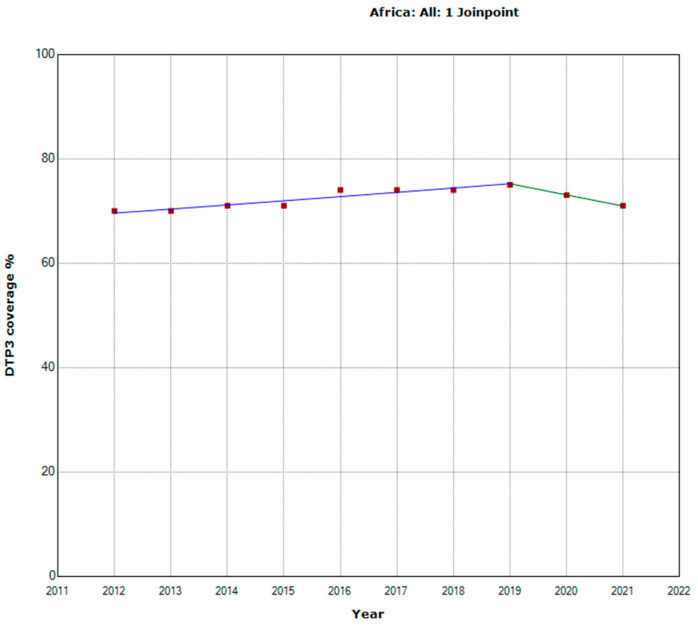
Third DTP doses vaccination rate trends in Africa (2012–2021) indicating joinpoints.

From 2012 to 2021, vaccination rates with the third dose of DTP in Africa experienced an annual percentage change (APC) of 0.5%. A joinpoint was detected in 2019. From 2012 to 2019, the vaccination rate had an increasing trend. From 2020 onwards, a decreasing trend began. From 2019 onwards, the APC was negative, with an annual decrease of −2.9%, although this change is not statistically significant due to the short period (Table 1, Figure 1).

**Figure 2:**
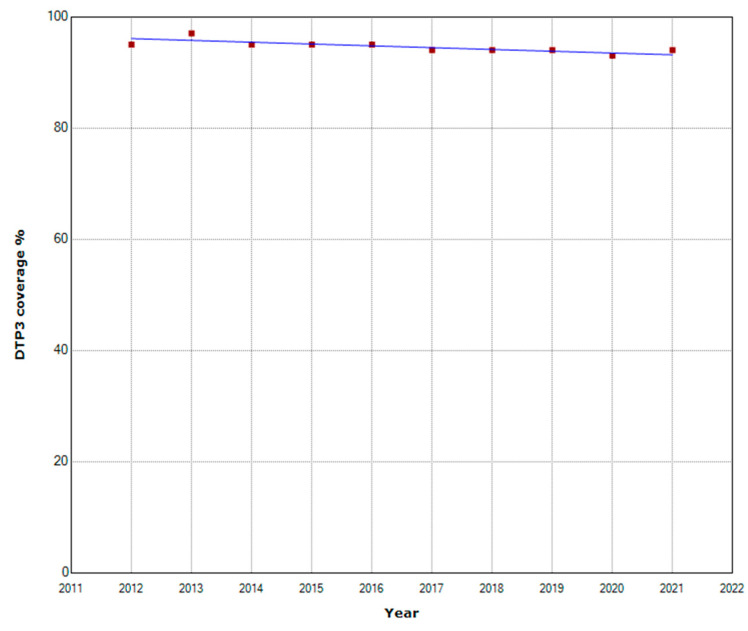
Third DTP doses vaccination rate trends (2012–2021): North Africa.

The joinpoint regression did not show any joinpoint in North Africa during 2012–2021 (Table 2, Figure 2). During the whole period, the APC was slightly negative −0.3%.

**Figure 3:**
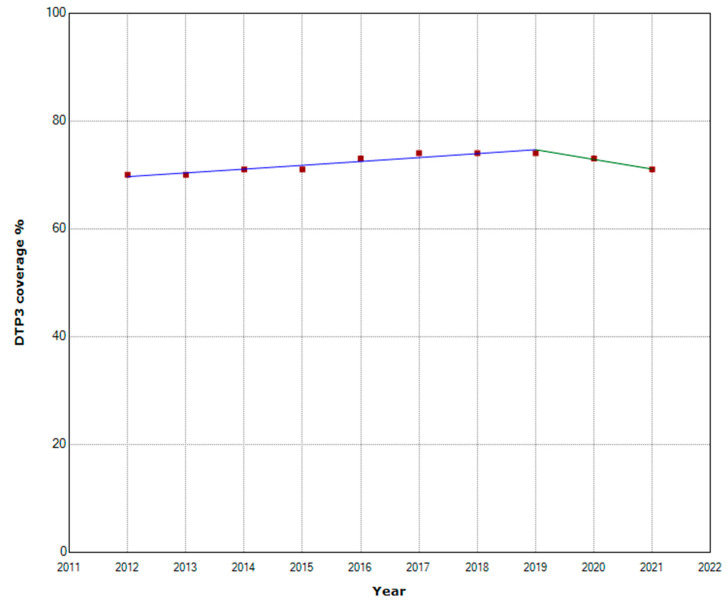
Third DTP doses vaccination rate trends in Sub-Saharan Africa (2012–2021) indicating joinpoints.

In Sub-Saharan Africa, one joinpoint was detected in 2019. From 2012 to 2019, the vaccination rate increased with an APC of 1%. This increasing trend was interrupted in 2020 when a decreasing trend began with an APC of −2.4% (Figure 3).

**Figure 4:**
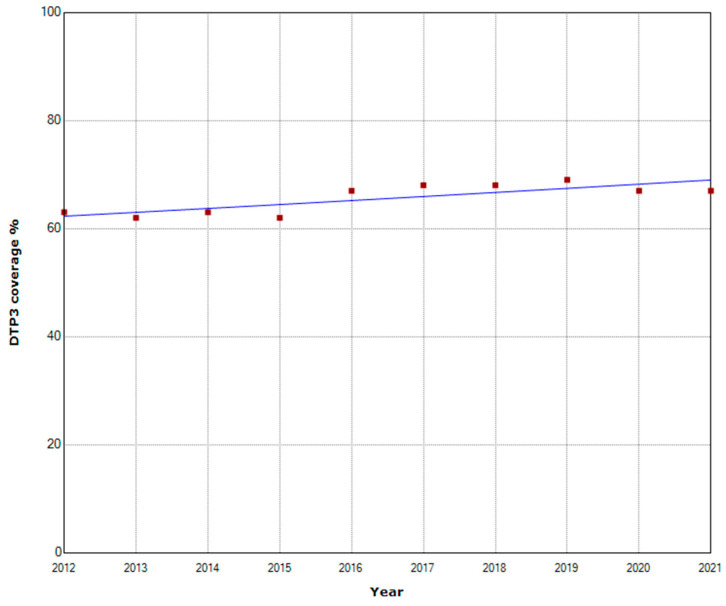
Third DTP doses vaccination rate trends (2012–2021): West and Central Africa.

Regional analysis in Sub-Saharan Africa found no joinpoint in West and Central Africa. Throughout the whole period, coverage increased with an APC of 1.1% (Figure 4).

**Figure 5:**
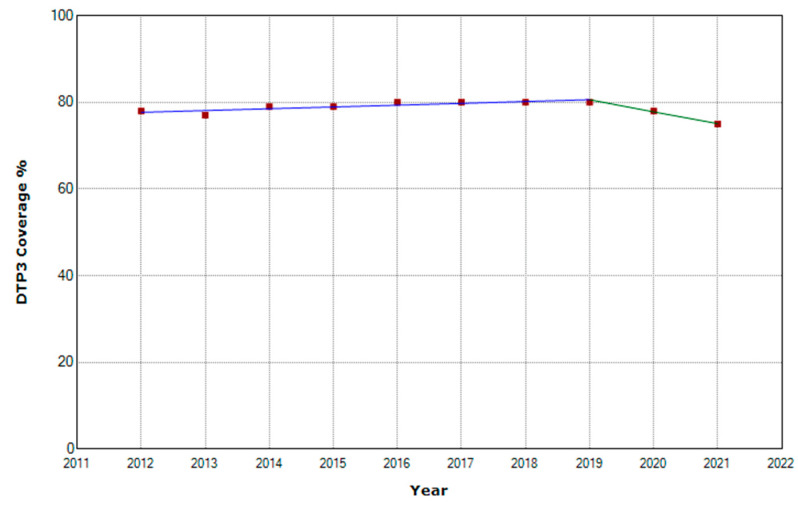
Third DTP dose coverage (2012–2021) indicating joinpoints in Eastern and Southern Africa.

In the Eastern and Southern Africa regions, there was a joinpoint in 2019. The APC was positive until 2019 and negative from 2020, with an APC of −3.5% (Figure 5).

The absolute decrease in vaccine coverage in Africa between 2019 and 2021 was −4%. The coverage remained the same in North Africa, while a more significant decrease occurred in Eastern and Southern Africa at −5% (Table 3).

Table 4 presents the DTP third dose coverage in 2019 and 2021. The average country decrease was −3.19 (SD = 6.598), *p* < 0.001. Figure 3 presents the map of Africa, indicating the absolute differences in vaccine coverage between 2012 and 2021. Vaccination coverage declined in 26 countries, representing 51% of the countries. In countries where vaccination coverage decreased, the absolute decrease had a mean of −7.58 (SD = −6.64).

There were 14 countries where vaccination coverage was maintained. There were 11 countries with increased vaccination coverage. In countries where vaccination coverage decreased, the absolute decrease had a mean of −7.58 (SD = −6.64) (Table 4) (Figure 5).

**Figure 6:**
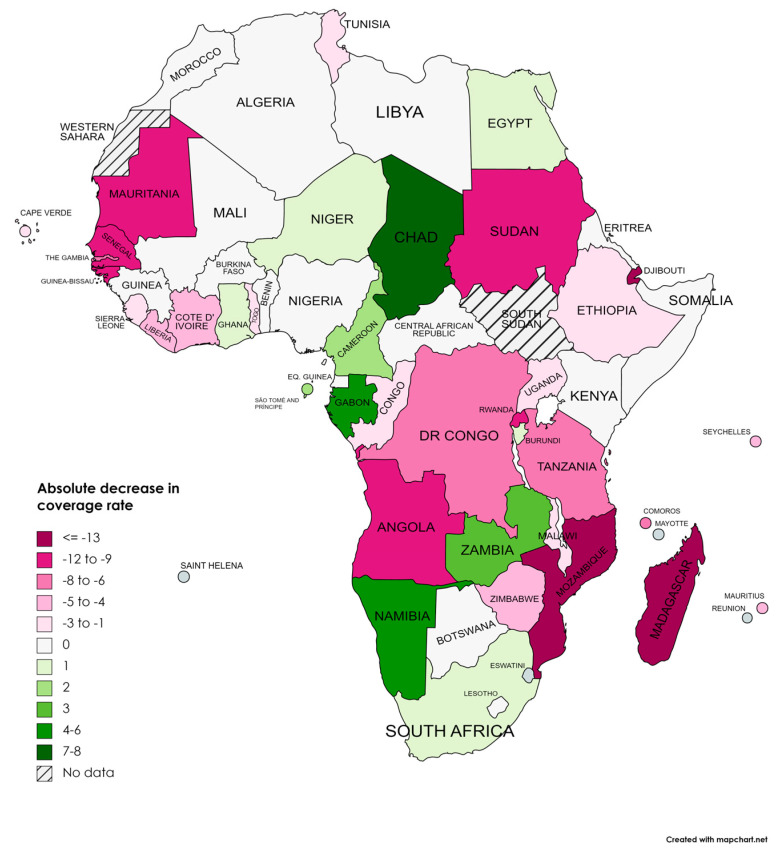
Absolute Changes in DTP Vaccination Coverage (%) Across African Countries between 2019 and 2021.

**Figure 7:**
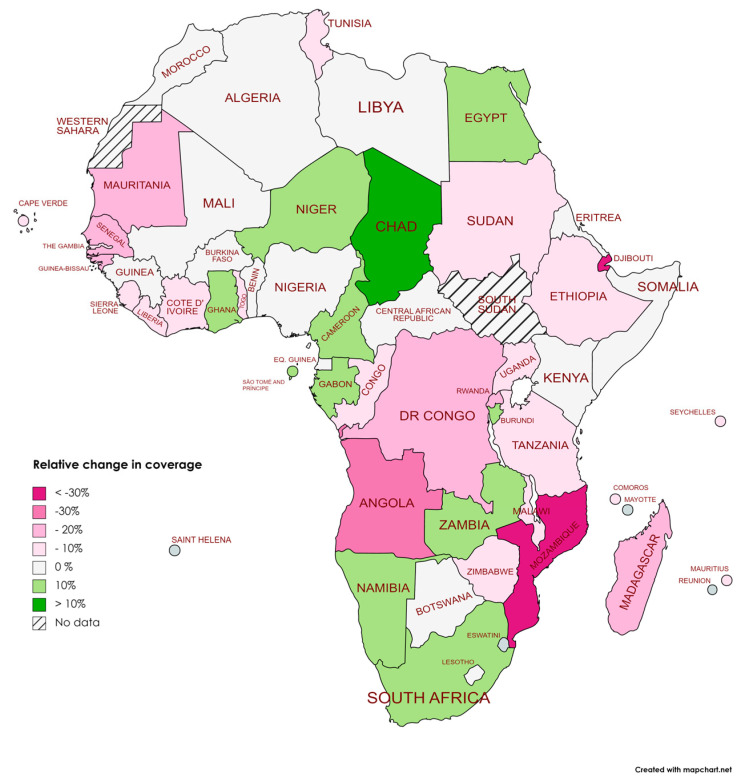
Relative changes in DTP3 vaccination coverage (%) across African countries between 2019 and 2021.

The relative coverage decrease in Africa between 2019 and 2021 was −5.33%. The more significant relative decrease was in Eastern and Southern Africa, with a relative decrease of −6.25% (Table 3). Mozambique and Djibouti were the countries with a large relative decrease in coverage, with reductions of −30.7% and −30.6%, followed by Angola (−21.1%) and Madagascar (−19.1%). Despite the crisis, Chad had a relative increase of more than 10%. Other countries, such as Cameroon, Zambia, Namibia, and Gabon, substantially increased their coverage (Table 4) (Figure 6 and Figure 7).

In North Africa, in Egypt, DTP3 vaccine coverage slightly increased by 1% between 2019 and 2021. In three countries of North Africa, Algeria, Libya, and Morocco, the coverage remained the same, while it decreased slightly in Tunisia and more significantly in Mauritania, where absolute coverage decreased by 12%.

**Figure 8:**
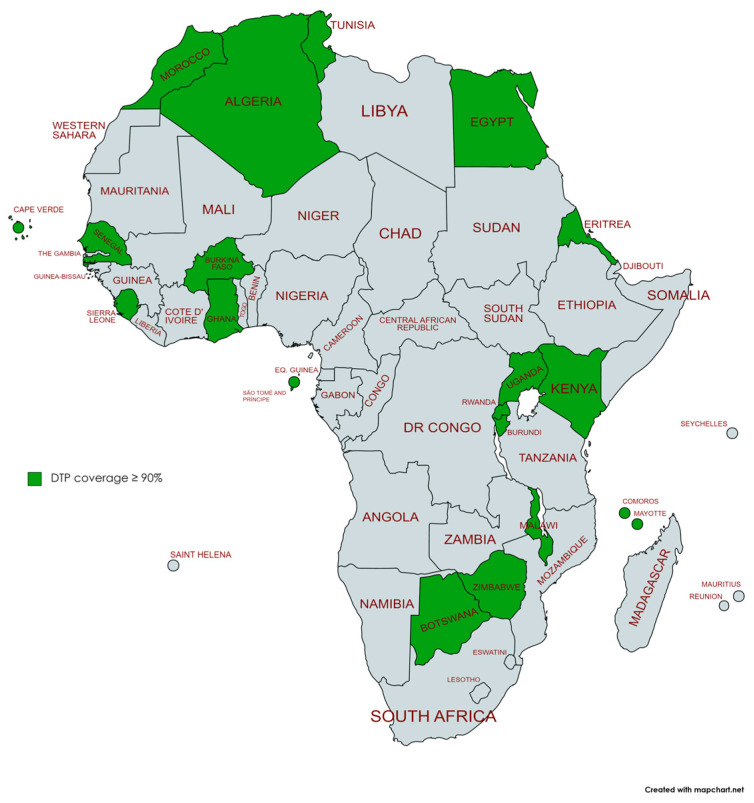
African countries meeting the WHO 90% DTP3 vaccination coverage target 2019.

**Figure 9:**
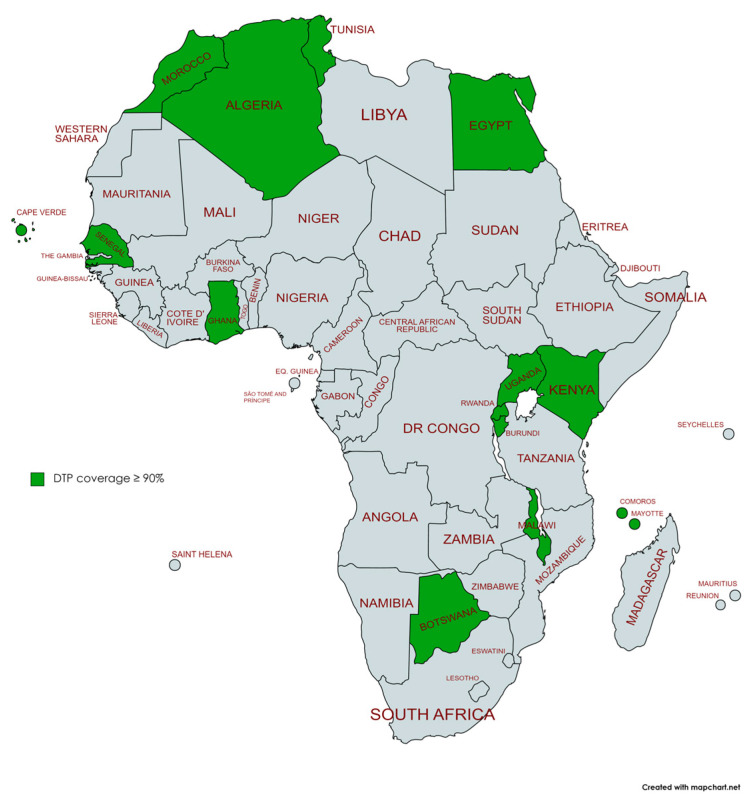
African countries meeting the WHO 90% DTP3 vaccination coverage target 2021.

By 2019, 21 African countries had reached the WHO target of 90% coverage for the third dose of DTP (Botswana, Burkina Faso, Burundi, Cabo Verde, Comoros, Egypt, Eswatini, Eritrea, Ghana, Kenya, Malawi, Mauritius, Morocco, Rwanda, Sao Tome and Principe, Senegal, Seychelles, Sierra Leone, Sudan, Tunisia, Uganda, and Zimbabwe). Following the pandemic, by 2021, six of these countries (Burkina Faso, Eritrea, Eswatini, Sao Tome and Principe, Sierra Leone, and Zimbabwe) had dropped off this list (Figure 8 and Figure 9).

**Figure 10:**
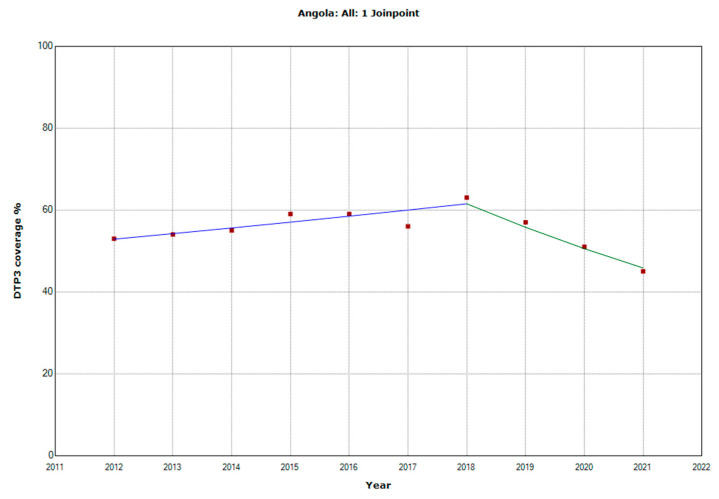
Third DTP dose coverage (2012–2021) indicating joinpoints in Angola.

**Figure 11:**
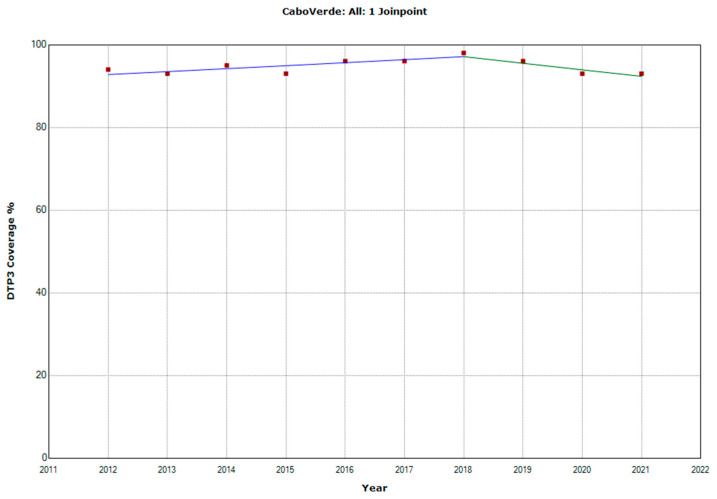
Third DTP dose coverage (2012–2021) indicating joinpoints in Cabo Verde.

**Figure 12:**
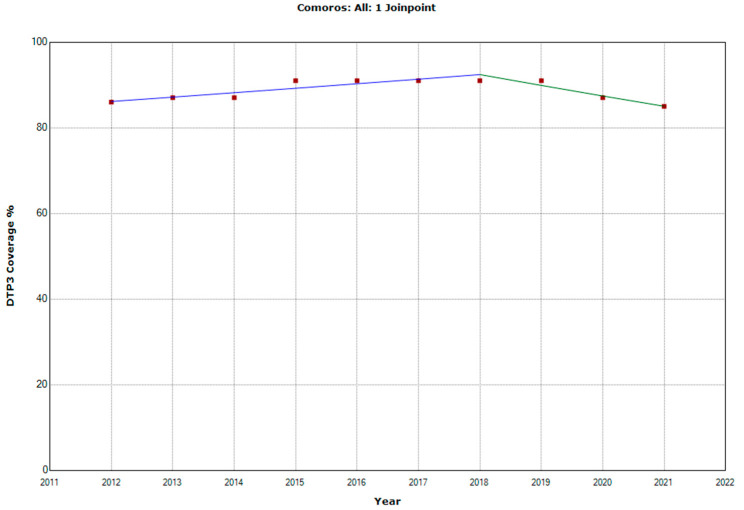
Third DTP dose coverage (2012–2021) indicating joinpoints in Comoros.

**Figure 13:**
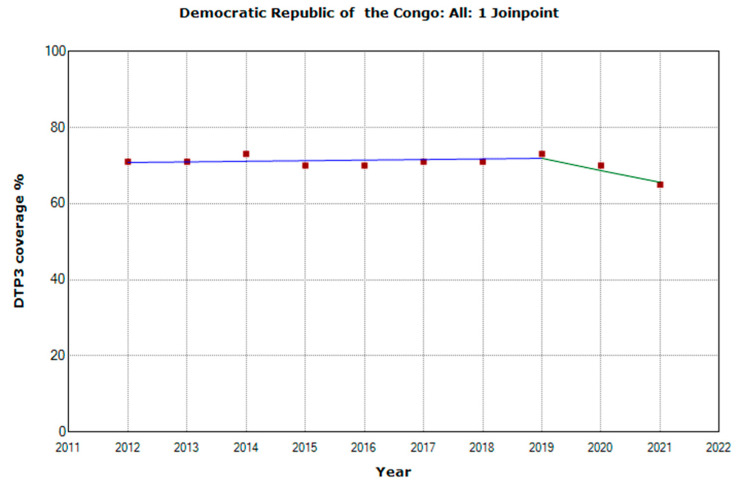
Third DTP dose coverage (2012–2021) indicating joinpoints in the Democratic Republic of Congo.

**Figure 14:**
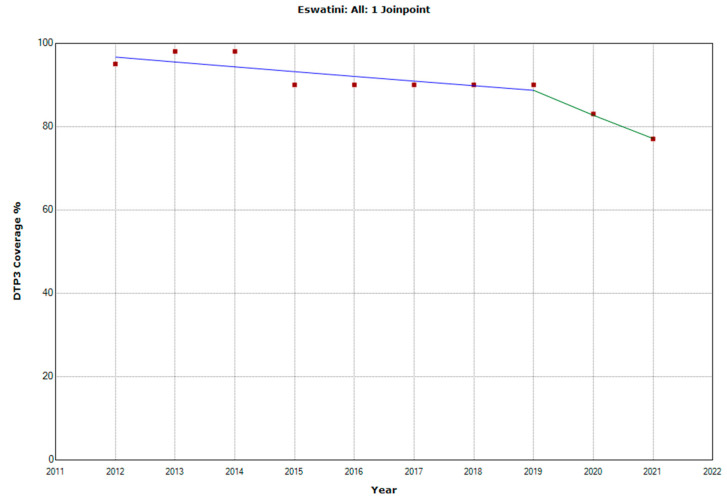
Third DTP dose coverage (2012–2021) indicating joinpoints in Eswatini.

**Figure 15:**
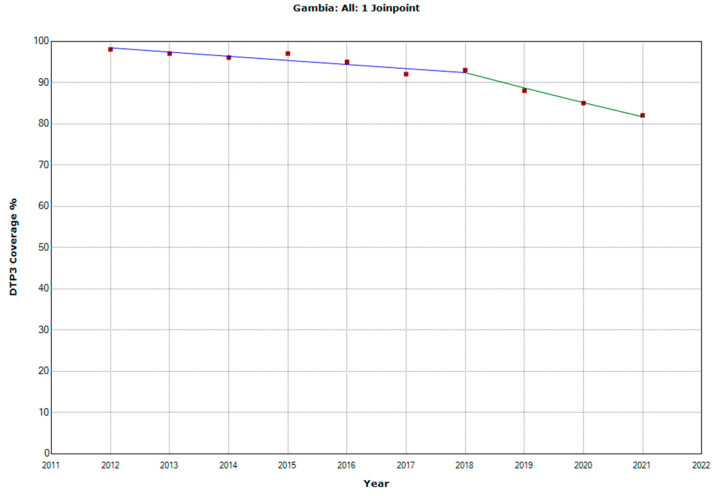
Third DTP dose coverage (2012–2021) indicating joinpoints in The Gambia.

**Figure 16:**
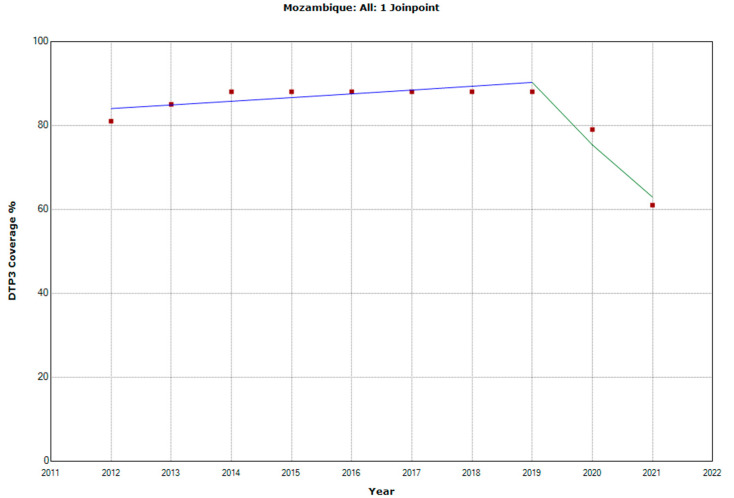
Third DTP dose coverage (2012–2021) indicating joinpoints in Mozambique.

**Figure 17:**
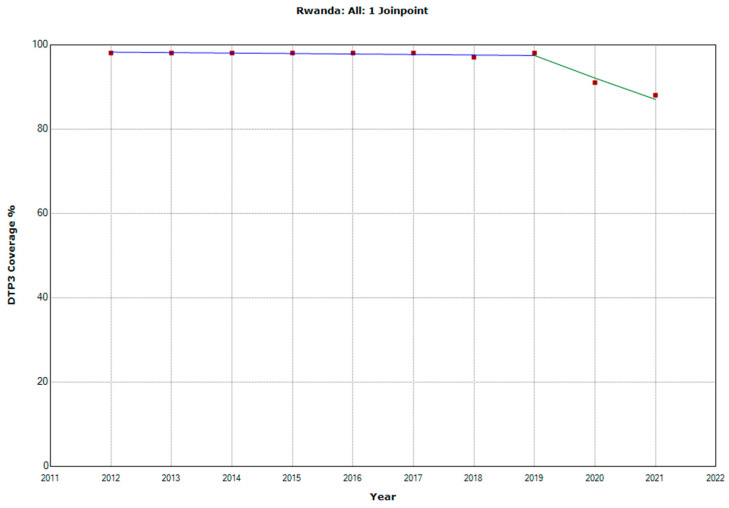
Third DTP dose coverage (2012–2021) indicating joinpoints in Rwanda.

**Figure 18:**
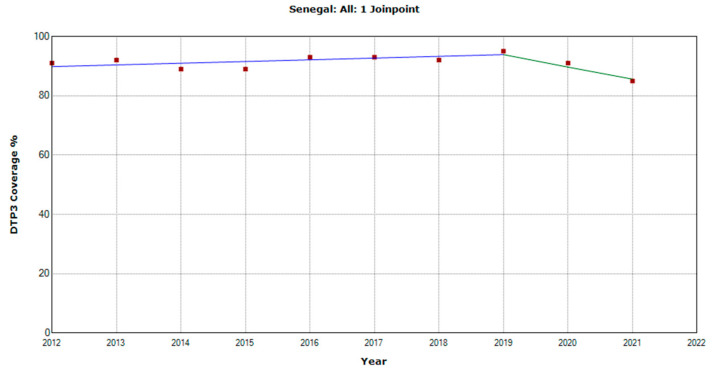
Third DTP dose coverage (2012–2021) indicating joinpoints in Senegal.

**Figure 19:**
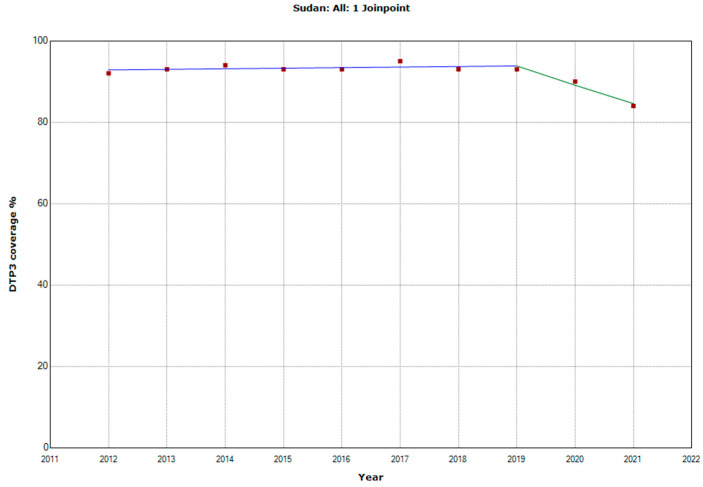
Third DTP dose coverage (2012–2021) indicating joinpoints in Sudan.

There were 10 countries with a joinpoint close to 2019: Angola, Cabo Verde, Comoros, Democratic Republic of the Congo, Eswatini, The Gambia, Mozambique, Rwanda, Senegal, and Sudan (Table 5, Figure 10, Figure 11, Figure 12, Figure 13, Figure 14, Figure 15, Figure 16, Figure 17, Figure 18 and Figure 19). In four countries (Angola, Cabo Verde, Comoros, and The Gambia), there was a joinpoint in 2018 (95% CI 2017–2019), while, in six countries, the joinpoint was in 2019 (95% CI 2018–2020). In all these countries, there was a decrease in the APC after the joinpoint, ranging from −1.7% in Cabo Verde to −16.5% in Mozambique.

## 4. Discussion

This study utilized UNICEF data to evaluate DTP vaccination trends across Africa. UNICEF is widely recognized for collecting and disseminating high-quality child health indicator data, including vaccination rates. Their standardized methodology ensures consistency in data collection, processing, and reporting across countries and periods, facilitating comparative analysis of trends. Despite potential limitations such as data availability and possible reporting discrepancies, the extensive coverage of UNICEF data, encompassing most African nations, provides a reliable and valuable basis for our analysis.

However, despite UNICEF’s standardized data collection and reporting methodology, data quality and accuracy discrepancies may emerge due to variations in reporting practices, data management, and healthcare systems across different countries. These discrepancies could affect our analysis and conclusions [35]. Moreover, the aggregated nature of UNICEF data at national and regional levels might obscure variations in vaccination trends within countries, particularly in areas marked by disparities in healthcare access or sociodemographic factors.

It is important to mention that the data was last updated in 2021, excluding the most recent 2022 data. Consequently, the trends we observed might not fully encapsulate the ongoing impact of the COVID-19 pandemic on DTP vaccination rates in Africa [35]. Future studies accessing more recent data could provide further insight into these trends.

We employed various techniques, such as country comparisons between 2019 and 2021 of coverage and analysis of trends using the joinpoint. We revealed significant decreases in vaccine coverage across many countries. We also showed that in Sub-Saharan Africa, a shift occurred in coverage trends, although this was not statistically significant due to the limited number of years analyzed.

The reduction in vaccine coverage due to COVID-19 has been a global issue, affecting other vaccines as well. Between January and December 2020, it is estimated that 30 million children missed doses of DTP3 and 27.2 million missed MCV1 doses [24]. Global coverage of DTP3 experienced a decrease of −5.81% between 2019 and 2021. The coverage rate declined from 86% in 2019 to 81% in 2020 [35]. In Latin America and the Caribbean, the coverage of DTP3 saw a decrease of −5.06% between 2019 and 2021, dropping from 79% in 2019 to 75% in 2020 [35]. On the other hand, Europe experienced a minimal decrease of −1.05% between 2019 and 2021, with coverage rates reducing from 95% in 2019 to 94% in 2020 [35].

Our study aimed to assess the impact of the COVID-19 pandemic on DTP vaccination trends in Africa. The results indicate that the pandemic has adversely affected vaccination coverage in various African countries, particularly Sub-Saharan Africa. The overall trend of DTP vaccination rates in Africa has shifted from a positive annual percentage change (APC) to a negative one, although the change is not statistically significant due to the short study period. The decrease in DTP coverage is a global issue. The Global coverage of the third dose of DTP (DTP3) fell from 86% in 2019 to 83% in 2021 [43]. There may be discrepancies between the number of African countries that experienced a significant decline in vaccination coverage between 2019 and 2021 (26 countries) and those in which a change in trend was detected that was smaller (only 10 of those 26 countries). This discrepancy is explained by the fact that although there has been a decrease in vaccination coverage rates, it is not yet of the magnitude to detect a trend change in some countries.

The decrease in vaccination rates can be attributed to several factors related to the COVID-19 pandemic, such as the diversion of healthcare resources towards managing the outbreak, lockdowns, and movement restrictions impeding access to vaccination services. Furthermore, fear of contracting the virus might have led to a reluctance among parents to bring their children to healthcare facilities for vaccination. A study in Africa found that the risks associated with suspension or vaccination outweigh the risk of contracting COVID-19 while receiving routine immunization services [44].

We found that DTP3 coverage remained relatively stable across most North African countries from 2019–2021, as depicted in Table 4. This observation warrants further exploration, as it contrasts with the 4% absolute decrease in vaccine coverage reported across Africa during the same period. Algeria, Libya, and Morocco demonstrated no absolute changes in their DTP3 vaccine coverage between 2019 and 2021. These findings may reflect the effectiveness of their national immunization programs and robust healthcare systems, which allowed these countries to maintain their vaccination rates despite the challenges presented by the global COVID-19 pandemic. Egypt, on the other hand, observed a slight increase of 1% in vaccine coverage. This improvement might be attributed to concerted efforts by the Egyptian Government and international organizations to strengthen the national immunization services, particularly in rural and underserved areas [45,46].

Conversely, during this period, Tunisia and Mauritania experienced decreases in DTP3 vaccine coverage. The slight decrease of 1% in Tunisia might not be statistically significant, considering the high baseline coverage of 98% in 2019. In contrast, Mauritania’s absolute coverage decrease of 12% is substantial. This decrease may be attributed to the country’s ongoing health system challenges, including vaccine supply chain inefficiencies, which could have been exacerbated by the additional strain of the COVID-19 pandemic [3].

These findings highlight the need for tailored strategies to improve vaccine coverage, particularly in countries with decreasing trends. Continuous monitoring of immunization programs, addressing health system barriers, and community engagement are vital for maintaining and enhancing vaccine coverage in these settings.

A recent meta-analysis has found that the prevalence of incomplete immunization (failing to receive any vaccination doses) in Africa was 35.5%. The main risk factors were home birth, rural residence, lack of antenatal care visits, knowledge of immunizations, and maternal illiteracy [47].

Our study’s findings align with other research suggesting that the COVID-19 pandemic has disrupted essential health services, including vaccination programs, worldwide.

In Sub-Saharan Africa, the decreasing trend was more prominent, with an APC of −2.4%. This decrease is consistent with previous studies that reported disruptions in routine immunization services in the region due to the COVID-19 pandemic. The most significant decreases in vaccination coverage were observed in Eastern and Southern Africa, with a relative decrease of −6.25%. The interruption in the upward trend of vaccination rates in Sub-Saharan Africa is concerning, given the region’s already low vaccination coverage and higher burden of vaccine-preventable diseases.

Interestingly, despite the difficulties the pandemic posed, some nations, including Chad, Cameroon, Zambia, Namibia, and Gabon, were able to increase their vaccination coverage. This increase may be attributed to effective immunization strategies, targeted interventions, or increased government support for vaccination programs during the crisis. It is crucial to examine and learn from the experiences of these countries to improve immunization services in the face of future crises.

The implications of reduced vaccination coverage are significant, as it may lead to outbreaks of vaccine-preventable diseases such as diphtheria, tetanus, and pertussis. These outbreaks can strain already overwhelmed healthcare systems and further exacerbate the impact of the COVID-19 pandemic on public health in Africa. It is essential to address the decline in vaccination rates and implement strategies to ensure the continuity of immunization services.

Several factors may explain the change in vaccine coverage. Unvaccinated children in Sub-Saharan Africa are more likely to be born to parents with no formal education and come from poorer households. They are often found in urban areas with high illiteracy rates and face limited maternal education and media access. Maternal health-seeking behaviors play a role in reducing the likelihood of children being unimmunized. Country-level factors, such as high fertility rates, and community-level factors, such as high illiteracy rates, contribute to the prevalence of unvaccinated children [25]. A systematic analysis revealed that, in Sub-Saharan Africa, the primary factors contributing to incomplete vaccination were time limitations faced by caregivers, insufficient understanding about vaccination, the absence of vaccines or personnel at healthcare facilities, missed opportunities for vaccination, concerns regarding minor side effects, limited access to vaccination services, and the vaccination beliefs held by caregivers [48]. Multiple missed vaccination opportunities exist among different populations and geographical regions.

Addressing these factors through targeted interventions and policies is essential to improve immunization rates and ensure the health of these children. Geographical and economic factors, access to healthcare services, the grade of development of healthcare infrastructure, vaccine supply chain issues, sociocultural factors, political stability, and the government’s commitment to vaccination may modulate the impact of the COVID–19 pandemic on DTP vaccination coverage [49,50]. One issue to be considered is that of inequalities within countries. National coverage rates may have held steady or even increased during the pandemic, masking regions where they have fallen due to the pandemic. That happened in Cameroon, where the increase in vaccination coverage masked the impact of COVIC−19 on the vaccination of children in COVID–19 hotspot regions [51]. A study in Zimbabwe found that the prevalence of missed opportunities for vaccination was 9% among mothers with no education and 21% among educated mothers. In Gabon, the figures were 85% and 89% [52]. A metanalyses in Ethiopia found that effective strategies to increase vaccination coverage included promoting institutional delivery, reducing travel time to vaccination sites, encouraging antenatal care visits, educating mothers about immunization, providing information on the immunization schedule, targeting urban areas, and conducting postnatal household visits by healthcare providers [53]. In Somalia, the integration of human and animal vaccination campaigns has been shown to be an effective method of reaching nomadic and pastoralist communities of Somalia [54].

We can observe that the countries that dropped out of the list in 2021 of those achieving WHO’s DPT objectives are spread across different African regions. The decline in the number of countries reaching the target of 90% coverage for the third dose of DTP is observed across various African regions. It is essential to accurately assess the regional impact by comparing the countries that achieved the WHO target in 2019 and 2021. The decline in the number of countries reaching the target can be observed across multiple African regions. Another issue is that, even if vaccination programs improve and return to pre-pandemic levels, they might not do so to the extent required for catch-up immunizations, and this may take a long time [55]. Because of this delay, there may be an increased chance of outbreaks of diseases that could be prevented by vaccination [56].

The COVID–19 pandemic’s disruption has brought attention to the need for resilient health systems that can continue providing essential medical services in times of need. [57]. Efforts should be made to strengthen vaccination programs and improve coverage across the continent, particularly in the regions most affected by the decrease in vaccination rates [58]. Efficient planning, organization, and swift implementation are crucial for expediting the vaccine distribution process and ensuring a speedy recovery of vaccine coverage [59]. Collaboration between governments, healthcare providers, and international organizations are vital to ensure that progress in achieving vaccination targets is not lost [60] and to protect the health of African populations from vaccine-preventable diseases. New and strengthened partnerships are being forged to address these challenges and work towards achieving universal access to immunizations [61].

## 5. Conclusions

This study examined the repercussions of the COVID–19 pandemic on DTP3 vaccination trends in Africa from 2012 to 2021. The findings highlighted a negative annual percentage change (APC) of −2.9% starting in 2019, leading to an absolute decrease in vaccine coverage of −4% between 2019 and 2021. The most substantial relative reduction in coverage was observed in Eastern and Southern Africa, with a relative decline of −6.25%.

A regional analysis of Sub-Saharan Africa disclosed a joinpoint detected in 2019, marking an increasing trend until that year and a decreasing trend from 2020 onward. In Eastern and Southern Africa, the APC transitioned from positive to negative in 2020, showcasing an APC of −3.5%. Notably, in 10 countries (Angola, Cabo Verde, Comoros, the Democratic Republic of the Congo, Eswatini, The Gambia, Mozambique, Rwanda, Senegal, and Sudan), the joinpoint regression detected a trend alteration.

The number of African nations achieving the World Health Organization’s 90% coverage goal for the third DTP dose suffered a significant setback of 22.7%, plummeting from 22 countries in 2019 to 17 in 2021. This decline provides compelling evidence of the detrimental impact of the COVID-19 pandemic on DTP vaccination trends in Africa, as half of the countries surveyed experienced a contraction in vaccination coverage.

## Figures and Tables

**Table 1:** Joinpoint analysis for the third doses DTP vaccination rates in Africa, 2000–2017.

Periods	Years	APC (95% CI)	*p*
Total Period	2012–2021	0.5 (0; 1.1)	0.052
Period 1	2012–2019	1.1 (0.7; 1.6)	0.001
Period 2	2019–2021	−2.9 (−6.9; 1.3)	0.134

**Table 2:** Joinpoint analysis for Regional 3rd DTP dose coverage in Africa, 2012–2021.

Periods	Years	APC (95% CI)	*p*
North			
Total Period	2012–2021	−0.3 (−0.4; −0.2)	0.001
Sub-Saharan Africa			
Total Period	2012–2021	0.5 (0; 1.0)	0.053
Period 1	2012–2019	1.0 (0.6; 1.4)	0.001
Period 2	2020–2021	−2.4 (−5.2; 1.4)	0.078
West and Central Africa			
Total Period	2012–2021	1.1 (0.5; 1.8)	0.004
East and South Africa			
Total Period	2012–2021	−0.6 (0; 0.5)	0.835
Period 1	2012–2019	0.5 (0.2; 0.8)	0.006
Period 2	2020–2021	−3.5 (−6.3; −0.5)	0.029

**Table 3:** Absolute and relative changes in DTP vaccination coverage (%) across African regions between 2019 and 2021.

Region	2019	2021	Absolute Changes	Relative Changes
Africa	75	71	−4	−5.33%
West and Central Africa	69	67	−2	−2.90%
Eastern and Southern Africa	80	75	−5	−6.25%
Sub-Saharan Africa	74	71	−3	−4.05%
North Africa	94	94	0	0.00%

**Table 4:** Absolute and relative changes in DTP vaccination coverage (%) across African countries between 2019 and 2021.

Country	2019	2021	Absolute Changes	Relative Changes	*p* *
Algeria	91	91	0	0.0%	ns
Angola	57	45	−12	−21.1%	<0.001
Benin	76	76	0	0.0%	ns
Botswana	95	95	0	0.0%	ns
Burkina Faso	91	91	0	0.0%	ns
Burundi	93	94	1	1.1%	ns
Cabo Verde	96	93	−3	−3.1%	<0.001
Cameroon	67	69	2	3.0%	<0.001
Central African Republic	42	42	0	0.0%	ns
Chad	50	58	8	16.0%	<0.001
Comoros	91	85	−6	−6.6%	<0.001
Congo	79	77	−2	−2.5%	<0.001
Côte d’Ivoire	81	76	−5	−6.2%	<0.001
Democratic Republic of the Congo	73	65	−8	−11.0%	<0.001
Djibouti	85	59	−26	−30.6%	<0.001
Egypt	95	96	1	1.1%	<0.001
Equatorial Guinea	53	53	0	0.0%	ns
Eritrea	95	95	0	0.0%	ns
Ethiopia	68	65	−3	−4.4%	<0.001
Eswatini	90	77	−13	−14.4%	<0.001
Gabon	70	75	5	7.1%	<0.001
The Gambia	88	82	−6	−6.8%	<0.001
Ghana	97	98	1	1.0%	<0.001
Guinea	47	47	0	0,0%	ns
Guinea-Bissau	78	67	−11	−14,1%	<0.001
Kenya	91	91	0	0.0%	ns
Lesotho	87	87	0	0.0%	ns
Liberia	70	66	−4	−5.7%	<0.001
Libya	73	73	0	0.0%	ns
Madagascar	68	55	−13	−19.1	<0.001
Malawi	95	93	−2	−2.1%	<0.001
Mali	77	77	0	0.0%	ns
Mauritania	80	68	−12	−15.0%	<0.001
Mauritius	96	92	−4	−4.2%	<0.001
Morocco	99	99	0	0.0%	ns
Mozambique	88	61	−27	−30.7%	<0.001
Namibia	87	93	6	6.9%	<0.001
Niger	81	82	1	1.2%	<0.001
Nigeria	56	56	0	0.0%	ns
Rwanda	98	88	−10	−10.2%	<0.001
Sao Tome and Principe	95	97	2	2.1%	<0.001
Senegal	95	85	−10	−10.5%	<0.001
Seychelles	99	94	−5	−5.1%	<0.001
Sierra Leone	95	92	−3	−3.2%	<0.001
Somalia	42	42	0	0.0%	ns
South Africa	85	86	1	1.2%	<0.001
Sudan	93	84	−9	−9.7%	<0.001
Tanzania	89	81	−8	−1.2%	<0.001
Togo	84	83	−1	−3.4%	<0.001
Tunisia	98	97	−1	−1.0%	<0.001
Uganda	93	91	−2	−2.2%	<0.001
Zambia	88	91	3	9.0%	<0.001
Zimbabwe	90	86	−4	0.0%	<0.001

ns = no significant * Chi-square Test.

**Table 5:** Joinpoint analysis 3^rd^ DTP dose coverage in Africa, 2012–2021.

Country	Years	APC (95% CI)	*p*
Angola			
Period 1	2012–2018	2.6 (1.3; 4.1)	0.013
Period 2	2018–2021	−9.3 (−14.4; −6.1)	0.007
Cabo Verde			
Period 1	2012–2018	0.8 (0.3; 1.2)	0.006
Period 2	2018–2021	−1.7 (−3.2; −0.1)	0.040
Comoros			
Period 1	2012–2018	1.2 (0.6; 1.8)	0.004
Period 2	2018–2021	−2.7 (−4.6; −0.8)	0.014
Democratic Republic of the Congo			
Period 1	2012–2019	0.2 (−0.5; 1.0)	0.489
Period 2	2019–2021	−4.5 (−9.5; 0.8)	0.079
Eswatini			
Period 1	2012–2019	−1.2 (−2.6; 0.2)	0.078
Period 2	2019–2021	−6.8 (−15.7; 3.1)	0.134
The Gambia			
Period 1	2012–2018	−1.0 (−1.6; −0.4)	0.007
Period 2	2018–2021	−4.0 (−5.9; −2.1)	0.003
Mozambique			
Period 1	2012–2019	1.0 (0.0; 2.0)	0.047
Period 2	2019–2021	−16.5 (−24.4; −7.8)	0.006
Rwanda			
Period 1	2012–2019	−0.1 (−0.2; 0.0)	0.063
Period 2	2019–2021	−5.5 (−7.4; −3.6)	0.001
Senegal			
Period 1	2012–2019	0.6 (−0.3; 1.6)	0.141
Period 2	2019–2021	−4.5 (−10.7; 2.1)	0.138
Sudan			
Period 1	2012–2019	0.1 (−0.2; 0.5)	0.294
Period 2	2019–2021	−5.1 (−8.6; −1.4)	0.017

## Data Availability

Data are available from UNICEF database.
